# Consumers vary in their attitudes and expectations about dietary fibre: analysis of answers to a pan-European online survey

**DOI:** 10.1017/S1368980026102298

**Published:** 2026-03-25

**Authors:** Véronique Azaïs-Braesco, Anne Lionnet, Matthieu Maillot, Marjukka Kolehmainen

**Affiliations:** 1 VAB-Nutrition, France; 2 Arista Cereal Technologies- Limagrain Ingredients, France; 3 MS-Nutrition, France; 4 Institute of Public Health and Clinical Nutrition, University of Eastern Finland, Finland

**Keywords:** Dietary fibre, Nutrition policy, Incentives, Consumer survey

## Abstract

**Objectives:**

Dietary fibre (DF) has known health benefits, but consumer intake remains below recommended levels. This survey aimed at gathering and structuring information about DF-related attitudes of European consumers, including motivations and barriers, as well as preferred incentives for increasing DF intake.

**Participants:**

Representative sample of 7247 subjects from seven countries.

**Setting:**

Online survey.

**Design:**

Participants completed a questionnaire focused on knowledge about DF, perceived intake and reactions to incentives. Hierarchical clustering analysis was used to define ‘clusters’ based on response profiles and ‘groups’ based on socio-demographic characteristics.

**Results:**

Consumers had a relatively good overall understanding of DF. However, responses to more detailed questions revealed knowledge gaps. Half of respondents said they consumed enough DF. Among proposed incentives for increasing intake, respondents preferred the labelling of fibre-rich products, then the inclusion of fibre in a wide variety of foods. Five answer clusters were identified: ‘committed consumers’ (sufficient DF intake, convinced of benefits), ‘sceptical’ (little DF-related knowledge, unconvinced), ‘informed consumers’ (good DF knowledge, insufficient intake), ‘helpless consumers’ (low intake, unclear about how to increase) and ‘resistant consumers’ (little concern and knowledge, rejection of all incentives). Socio-demographic groups displayed slight differences in response profiles (e.g. relative to the whole sample: white-collar workers tended to be ‘committed’ rather than ‘resistant’ consumers, and seniors tended to be ‘informed’ or ‘helpless’ rather than ‘sceptical’).

**Conclusions:**

This study helped define subpopulations of European consumers based on DF-related attitudes and behaviours. Socio-demographics somewhat explained these differences and should be considered when developing strategies for increasing DF consumption.

## Graphical abstract

**Figure:**
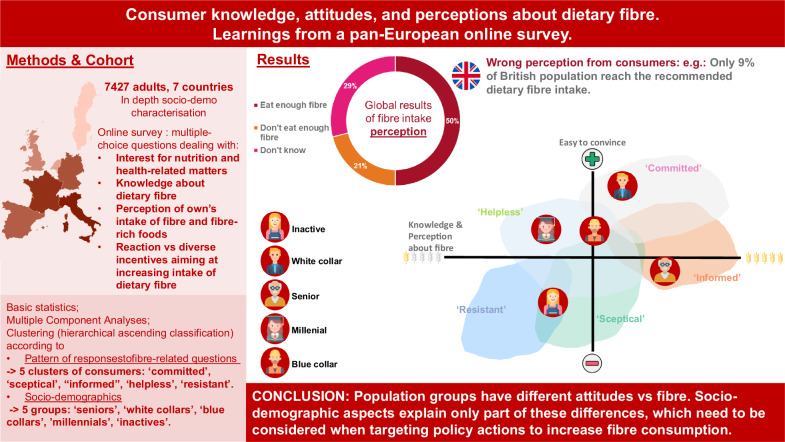

The health benefits of a fibre-rich diet are well established: both experimental and observational studies have demonstrated that dietary fibre (DF) can help prevent many common health conditions, such as obesity, type 2 diabetes, CVD and colorectal cancer^(1)^. Compared with low-fibre diets, high-fibre diets are also associated with reduced overall mortality^(2)^. The Global Burden of Disease Study concluded that in higher-income countries in 2017, a total of 256 disability-adjusted life years per 100 000 people were lost because of low DF consumption. Research suggests the optimal range of DF intake is 19–28 g/d^(3)^, a range recommended by most dietary guidelines worldwide. Additionally, a large meta-analysis of prospective and clinical trials^(4)^ suggests human health benefits could be achieved by recommending that refined grains be replaced with whole grains and that daily DF intake should be at least 25–29 g (with 30 g/d bringing additional benefits). Indeed, public health authorities have been promoting a DF intake of 25–35 g/d^(5,6)^, but surveys show mean DF intake is far from reaching this level in all countries^(7)^, creating a gap that threatens population health.

Public health messages have been seeking to increase the dietary intake of fibre-rich foods, most often focusing on fruits, vegetables, whole grains, nuts or seeds. Five-a-day campaigns have been around for many years, but the data suggest that only one of every eight Europeans above the age of 15 is consuming at least five daily portions of fruits and vegetables^(8)^. Efforts are also been made to encourage people to choose whole-grain over refined cereal products^(9)^. While the scientific community has developed an agreed-upon definition of whole grain with a view to food labelling^(10)^, consumer DF intake remains well below recommended levels in most countries^(3,7)^, in adults as in children^(11)^ or elderly^(12)^. Thus, public health campaigns aiming to increase fibre consumption directly or indirectly, through encouraging intake of fibre-rich foods, have largely been unsuccessful. For example, in the UK, multiple initiatives have been launched to increase DF intake, but the latter remained relatively constant between 2008 and 2017^(13)^.

Among the hypotheses raised to explain such failures is the lack of personalisation of campaigns aiming to reach the whole population. Indeed, targeted interventions have often demonstrated a better efficacy than global messages intended for the whole population, although improvements are often of limited magnitude. A meta-analysis of four randomised controlled trials found that daily DF intakes were around 2 g higher in subjects exposed to face to face or phone individual sessions of dietary advice compared with subjects with no or global interventions^(14)^. Other studies found that DF intake was increased more following tailored messages or personalised dietary advice compared with general dietary advice^(15,16)^. Although personalisation appears a relevant approach, scale-up of methods derived from lab-set trials carried out on a limited number of subjects to large populations appears questionable. Indeed, individual approaches are difficult and costly to implement; furthermore, they require subject’s willingness to participate and may thus miss the non-volunteers, who are often the most in need of help.

In this study, we hypothesised that the behaviour *v*. increasing DF intake will differ across consumers according to their socio-demographics but also to their interest for nutrition and health and to their knowledge, perceptions and attitude towards DF. Characterising such differences could help identify the needs of different consumers and inform the development of customised messaging, communication tools, food products and appropriate social marketing. We thus carried out a survey in seven European countries to gather information about consumers’ relationship with DF, including their level of nutritional understanding, their motivations for and barriers to DF intake and their preferred incentives for increasing DF intake. We also sought to investigate whether consumer knowledge, perceived intake, attitudes and expectations *v*. DF differed among groups within populations

## Methods

### Study population

An online survey was conducted in seven countries—Germany, France, the UK, Spain, Italy, the Netherlands and Sweden—from 15 November to 30 November 2021. The survey company Toluna selected the participants from its national panels, sent out the questionnaire and collected the raw answers. To obtain a representative sample of the national populations in these seven countries, participants were recruited using quota sampling. Country-specific quotas were designed for age, gender, region of residence and income. Country-specific targets for these quotas were defined using Eurostat statistics^(17)^. Once individuals have been recruited, their quota characteristics were verified by asking questions about socio-demographic characteristics (see below). The distribution obtained for the quota variables was then compared with the distribution of the Eurostat population. We then apply the calibration algorithm in order to adjust the sample so that the ‘margins’ (i.e. the averages per group) are similar, for each country, between the sample and the total population. The set of answers of each individual is attributed a calibration weight that corrects possible quota deviations during recruitment, based on respondents’ socio-demographic characteristics. As a result, the weighted averages calculated are representative of population averages.

Quality control measures included trap questions, detecting speeders (i.e. participants who took less than half of the median time to answer the full questionnaire) and detecting straight liners (i.e. participants who give the same answer to all questions).

### Questionnaire

#### Demographics

Respondents were asked for their sex, age (six age groups), region of residence, type of household (number of household members, presence of children), profession, current work situation (student, working, inactive and retired), level of education based on the International Standard Classification of Education (ISCED) framework (ISCED 0 to 2: pre-primary to lower secondary, ISCED 3 to 4: upper secondary to post-secondary non-tertiary and ISCED 5 to 8: tertiary) and income (three country-specific categories: low, medium and high).

#### Dietary fibre – knowledge, perceived intake and attitudes

The quantitative questionnaire used in this study was designed to last around 15 min in total. It was the product of a 6-day online qualitative forum held in February 2021; each day, there were over sixty respondents from three countries. During this preliminary step, we identified themes of interest and established the appropriate language level. The questionnaire was made up of close-ended questions focused on four thematic areas: the respondent’s degree of nutritional concern (two questions), knowledge about DF (sources, types, health benefits and ways to recognise fibre-rich foods), perceived intake of DF and fibre-rich food categories and incentives that would encourage them to consume larger quantities of fibre-rich foods. For some questions, there were several choices whose order was randomised for each respondent and who was asked to rank their top three choices. For some questions, outlier responses were proposed (e.g. ‘fats, oils and butter’ or ‘dairy’ as foods that contain the most DF). Questions were translated from English into each country’s official language by native speakers. The full text for the questionnaire is in the online supplementary material, Supplemental (Table S1), along with details on the few recoding operations used to simplify the data.

### Statistical analyses

We employed a statistical weighting procedure to ensure that each of the seven countries had a sample that was representative of its national population. The weights were calculated by calibrating the sample based on the recruitment quotas. The full sample was also calibrated based on the total population (i.e. from all seven countries), using the countries’ population sizes.

Respondent socio-demographics and knowledge, perceived intake and attitudes related to DF were described using the survey-weighted percentages for each country and the total study population. A Khi-2 test was conducted to determine whether there were country-specific patterns in socio-demographics or DF-related responses. The alpha level was 5 %.

Two independent clustering analyses were conducted. The first analysis was performed using the responses to the DF-related questions and helped identify and characterise different response profiles. The second analysis was performed using the socio-demographic variables and revealed patterns for respondents in particular socio-demographic groups. In both cases, a multiple correspondence analysis (MCA) was carried out, followed by a hierarchical clustering analysis (HCA) focused on the MCA’s principal components. MCA is a valuable tool for exploring and visualising the relationships among categorical variables in complex datasets. It aims to reduce the dimensionality of the data by transforming the original categorical variables into a lower-dimensional space of orthogonal dimensions called ‘principal component’ or ‘dimension.’ The number of principal components was determined by looking at the screen plot, which expresses the contribution of each principal component to total data variability. The HCA grouped participants with similar responses. The number of clusters was determined using the Ward criterion^(18)^, which minimises the amount of within-cluster variation, as well as a visual inspection of the dendrogram. The MCA and HCA were conducted with the *FactoMineR* package in R. To interpret the meaning of each cluster of answers, a Khi-2 test was computed between each fibre-related question and the cluster variable. The same analysis was done with the socio-demographic variables, resulting in socio-demographic groups.

The relationships between the response clusters and socio-demographic groups were analysed using a contingency table. A Khi-2 test was performed to ascertain response cluster and socio-demographic group independence. To better interpret the relationship, column percentages have been calculated, showing the repartition among the clusters of answers within each socio-demo clusters, and among the whole sample.

To obtain further information, a series of MCA were conducted in which the active variables were the socio-demographic groups and the responses to a subset of DF-related questions. The results were interpreted based on the first two principal components of the analyses.

All the statistical analyses were conducted using R version 4.3.3. and R studio 2023.12.1.

### Description of the study population

#### Socio-demographics

A total of 7670 respondents from seven countries filled out the questionnaire. The results for 243 respondents (3 %) were eliminated following quality control. The remaining 7427 respondents were the study population. The percentages of men and women were 48 % and 52 %, respectively. The percentage of younger people (18–34 years old) was 25·4 %, while the percentages of middle-aged people (34–54 years old) and older people (> 55 years old) were 35·8 % and 38·8 %, respectively. A total of 43·5 % of the respondents had children (> 18 years old) in their households. Within the study population, 15·8 % of respondents had a low level of education (ISCED 0–2), 53·2 % of respondents had an intermediate level of education (ISCED 3–4) and 30·9 % of respondents had a high level of education (ISCED 5–8). Income levels were low for 26·9 % of the study population, intermediate for 50·5 % of the population and high for 22·6 % of the population. Over half of the respondents (52·5 %) were actively working, 17 % were unemployed, 25·3 % were retired and 5·2 % were students. Considering their socio-professional category (i.e. independently from this current job situation), a third of the sample (32·9 %) declared to be employees, 18·3 % to be manual workers or blue collars, 34·8 % to be involved in intermediate or higher management and 13·8 % declared to be inactive. More detailed data and country-specific data are available in the online supplementary material, Supplemental (Table S2).

#### Concern about nutrition

Overall, respondents reported an intermediate degree of nutritional concern: around 50 % of people (range: 43·5 % in Spain to 53·5 % in France) scored their concern as a 2 or 3 (scale: 0–5). Fifteen percent of respondents were not concerned about nutrition. A third of Germans scored their concern as a 0 or 1, while only 9 % of French people, 8 % of Italians and 2 % of Spanish people did the same. There were also country-specific differences in whether purchasing healthy foods motivated consumer shopping choices: this motivation was ranked number 1 by 32 % of all respondents, with a maximum of 41 % in Spain and a minimum of 21 % in the UK. The next two most common motivations were ‘buying tasty food products’ (ranked first by 18 % of respondents) and ‘satisfying hunger and basic needs’ (ranked first by 14 % of respondents).

#### Understanding of dietary fibre

Respondents generally lacked clarity about DF types. Bran was by far the best-known DF. Sugar was identified as an outlier response, but 26 % of respondents identified vitamins as a DF.

**Table 1.** Knowledge about dietary fibres

%	Yes	No	Do not know		Yes	No	Do not know
Is this a dietary fibre?				Is this a health benefit related to a high-fibre diet?			
Bran	74	8	18	Intestinal transit	80	7	13
Cellulose	34	26	40	Body weight	69	11	20
Resistant Starch	28	26	46	Gut microbiota	68	8	24
Vitamins	26	46	28	Blood cholesterol	62	11	27
Pectin	21	29	50	Blood sugar	57	14	29
FOS/GOS*	15	29	56	Immune system	56	15	29
Sugar	13	63	24	Muscle strength	43	23	34
Polyphenols	13	27	60	Reduced cognitive decline	41	16	43

* FOS, fructo-oligosaccharide; GOS, galacto-oligosaccharide.

Respondents expressed that DF have a wide range of health benefits, with intestinal transit being the most frequently cited. Even the outlier responses related to muscle strength and cognitive decline were selected by over 40 % of respondents (country-specific data: see online supplementary material, Supplemental Tables S3–1 and S3–2).

Using food labels was reported as a mean to identify fibre-rich foods by over 80 % of respondents (see online supplementary material, Supplemental Table S3–3); other means of identification included food texture (45 % of respondents), taste (31 % of respondents), food colour (20 % of respondents) and food smell (14 % of respondents).

#### Perceived intake of dietary fibre and fibre-rich food groups

Half of the respondents believed they consumed enough DF. This figure was generally consistent across countries, although the Italians and the Dutch were more confident about their intake (65 % and 68 %, respectively). Overall, 29 % of respondents believed they did not consume enough fibre, and 21 % did not know. Over 40 % of respondents reported that they had a high intake of fruits and vegetables (45 %), nuts and seeds (45 %), legumes (41 %) and cereal-based products (55 %). More respondents reported a high intake of whole-grain cereals (32 %) than of non-whole-grain cereals (22 %). More data for this section can be found in the online supplementary material, Supplemental Materials (Table S4).

#### Preferred incentives for improving fibre intake (Table 2)

There was a preference for two incentives, which were among the top three choices for around 40 % of the respondents: (1) more clearly labelling high-fibre products (42 %) and (2) the presence of fibre in a wider range of foods (39 %). Seventeen percent stipulated that none of the proposed incentives would help them to increase their consumption of fibre-rich foods.

**Table 2.** Top three responses to the question ‘I would eat more fibre-rich foods if…’ (% of respondents)

Proposed incentive	1st	2d	3d	1 + 2 + 3
They were more clearly labelled ‘high-fibre products’	17	14	11	43
Fibres were present in a wider range of foods (bread, salads, pasta, sandwiches, flour, biscuits, ready-to-eat meals, etc.)	14	12	13	39
I had more or better information about their health benefits	11	10	10	31
They had the same price as equivalent low-fibre foods	11	10	10	30
High-fibre products were easy to find and easily available	8	10	11	29
I had proof that high-fibre foods contain only naturally occurring fibres	10	9	10	29
They had the same taste, texture or colour as low-fibre foods	7	8	8	23
They would not force me to change my eating habits	6	7	7	20
None of these would encourage me to eat more high-fibre foods*	13	15	17	–
I were more tolerant of high-fibre foods	4·7	3·6	3·9	12·2

* This incentive was no longer available for choice 2 when selected for choice 1 and the same was true for choice 3 when this incentive was selected for choice 2. Sum cannot be calculated.

With regard to incentive preferences, differences among countries were minimal. Compared with the whole study population, French people more frequently mentioned information about health benefits and the presence of naturally occurring fibres, whereas Italians less frequently mentioned the availability of high-fibre foods but more frequently mentioned the presence of naturally occurring fibres. The latter was not a preference for Dutch people. The availability and price of high-fibre foods were frequently mentioned by Spanish and Swedish people, while the greater availability of high-fibre products was often mentioned by people in Italy and the UK (country-specific data in online supplementary material, Supplemental Table S5).

### Multiple correspondence analyses and hierarchical clustering analyses

#### Clusters arising from fibre-related responses

**Figure 1.**
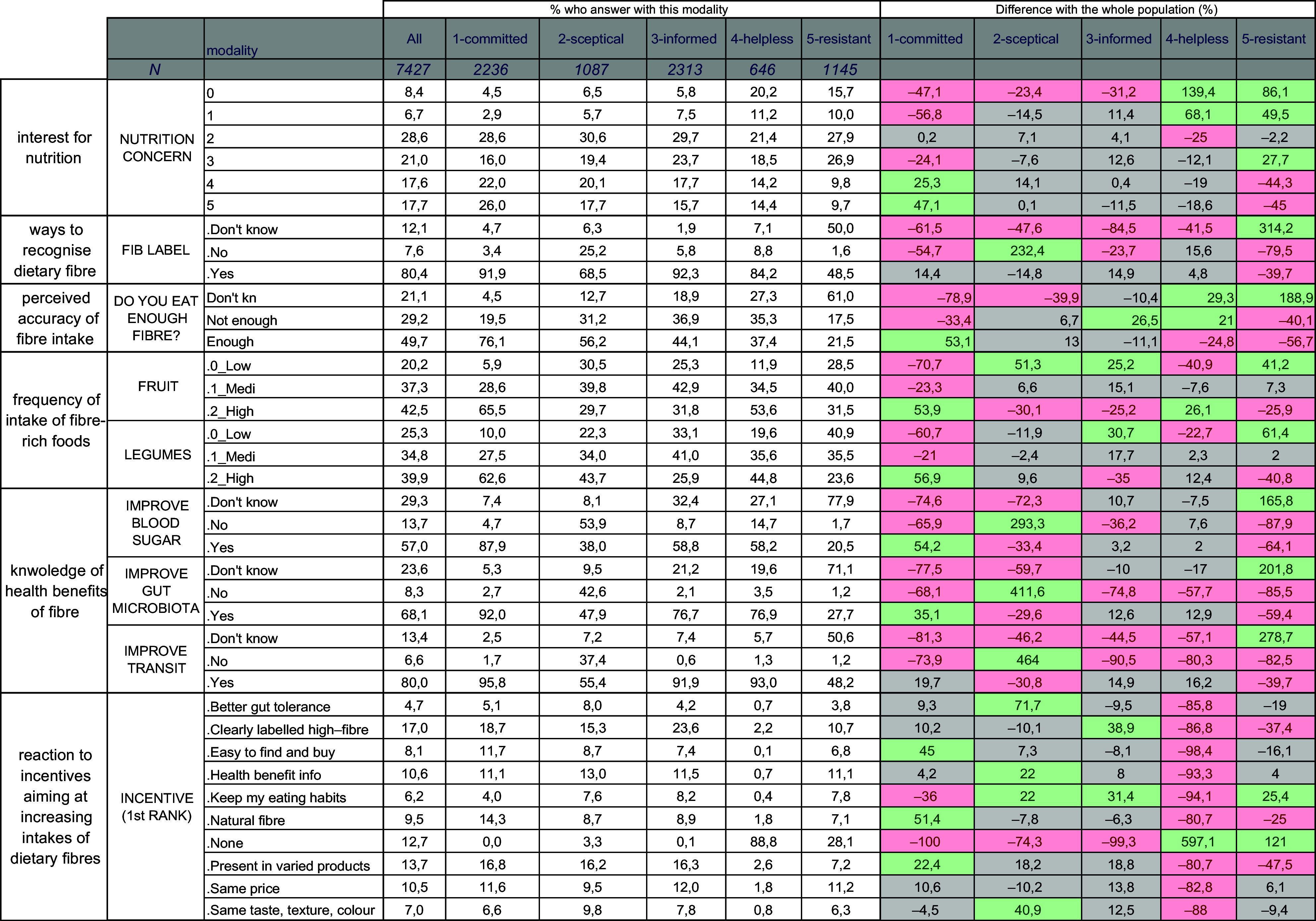
Responses to a subset of questions for the whole study population and each response cluster. The response clusters were identified using hierarchical clustering analysis. The five columns on the right show how each response cluster differed from the mean of the whole study population; differences greater than 20 % are in green (= this response had a greater frequency in the cluster than in the study population) or red (= this response had a lesser frequency in the cluster than in the study population).

The responses to the DF-related questions (without accounting for socio-demographics) were analysed in depth using MCA and HCA. The first four dimensions of the MCA were used in the HCA. Based on the dendrogram and the Ward criterion, five non-overlapping response clusters were identified (see online supplementary material, Supplemental Figure S1). Figure 1 displays the response type for the respondents in each cluster for a subset of questions. All the responses to all the questions can be found in Table S6. The differences in responses made it possible to characterise the clusters, notably by comparing a cluster’s responses with the responses for the whole study population.

*Cluster 1* contained 23·8 % of all respondents. This cluster contained people who expressed a high level of nutritional concern, who reported that they were eating enough fibre and who indicated that they frequently consumed fibre-rich foods. They had a very positive view of DF’s health benefits and even chose outlier responses (related to muscle strength or cognition). They more frequently used sensory properties (smell, taste, texture, etc.) rather than labels to identify fibre-rich foods. Among their preferred incentives were those focused on foods with naturally occurring fibres and practicality; they appeared open to changing their dietary habits. Overall, people in this cluster were confident in their knowledge (they seldom answered ‘I do not know’) and in their nutrition-oriented behaviour. At the same time, they remained open to further increasing their DF intake. Members of this cluster were referred to as the ‘committed consumers’.

*Cluster 2* contained 15·6 % of all respondents. People in this cluster generally gave responses that were reflective of those for the whole study population, except that they much more frequently denied that DF has any health benefits: 61 % did not believe that DF intake had positive effects on blood glucose, cholesterol metabolism, immunity or body weight and many also denied that DF intake had positive effects on intestinal transit (more than 35 %) or gut microbiota (41 %). People in this cluster more frequently responded that dairy, meat or fats were sources of DF and less often used labels to identify high-fibre foods. Among the incentives for increasing fibre intake, they more frequently mentioned their lesser tolerance of high-fibre foods and wanting foods with the same sensory properties, especially colour. Members of this cluster were referred to as ‘sceptical consumers’.

*Cluster 3* contained 31·7 % of all respondents. Again, people in this cluster generally gave responses that were reflective of those for the whole study population. However, they more frequently responded that they did not consume enough DF (37 % *v*. 29 % in the study population). They differed from cluster of sceptical in the following ways: they less frequently denied the health benefits of DF, they more frequently knew that dairy, fats and meat do not contain fibre and they more frequently used labels to identify fibre-rich foods (i.e. they less often use any sensory features). This response pattern appeared to be connected to their preferred incentive of better food labelling. Members of this cluster were referred to as ‘informed consumers’.

*Cluster 4* contained 16·5 % of all respondents. People in this cluster shared certain response characteristics with ‘informed’ consumers. However, they even more frequently reported that they did not consume enough DF and that they seldom consumed fibre-rich foods, especially cereal-based products, vegetables, legumes and nuts and seeds. They more frequently responded that they did not know how to identify fibre-rich foods and, although they were aware of the health benefits of DF, they wanted more information. They did not seem ready to change their eating habits, and price was an important incentive for them. Members of this cluster were referred to as ‘helpless consumers’.

*Cluster 5* contained 12·4 % of all respondents. People in this cluster reported a low level of nutritional concern and more frequently responded ‘I don’t know’ to a wide range of questions, including those related to DF intake (61 % *v*. 21 % in the study population), DF health benefits and ways of identifying fibre-rich foods. For example, 65 % responded that they did not know whether DF promoted intestinal transit, a figure that was 13 % for the whole study population; 58 % declared that they did not know whether labels could help identify fibre-rich foods. Furthermore, 39 % of the people in this cluster responded that none of the proposed incentives would help them increase DF intake, compared with 13 % of the whole study population. Members of this cluster were referred to as ‘resistant consumers’.

#### Socio-demographic groups and their relationship with fibre-related knowledge, perceived intake and attitudes

Similarly, clustering analysis was performed using the socio-demographic variables. HCA was performed using the first two components of the MCA, resulting in five groups of individuals who were similar in socio-demographic characteristics (see online supplementary material, Supplemental Figure S2; detailed description of groups in Table S7). In sections below, there is a detailed exploration of the relationships between these socio-demographic groups and the responses to DF-related questions that came out of the MCA.

##### ‘Senior’ group

**Figure 2.**
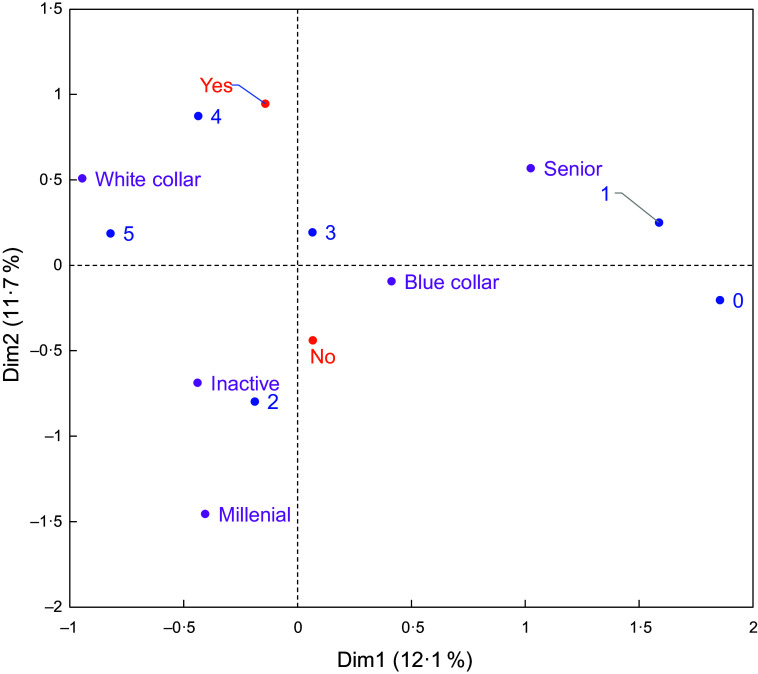
Multiple correspondence analysis using the socio-demographic groups (in purple), degree of nutritional concern (score range: 0–5; in blue), health as a major criterion in food purchases (yes/no; in orange). Along the horizontal axis, respondents are discriminated based on their level of nutritional concern. Those who expressed a high level of concern (score: 4–5) occur near the ‘white collar’ group, while those who expressed a low level of concern (score: 0–1) occur near the ‘senior’ group. Respondents who expressed an intermediate level of concern occur near the ‘blue collar’, ‘inactive’ and ‘millennial’ groups. Along the vertical axis, respondents are discriminated based on whether purchasing healthy foods was a major criterion in their shopping choices or not.

A quarter of respondents (24·7 %) were older people, and the majority were older than 65 years of age (84 %). The vast majority were living in a household without children (91 %) and were retired (95 %). It seems likely that the retirees responded to the question about their profession based on their job prior to retirement, which would explain why 0 % were ‘inactive’. This group contained slightly more French, German and Swedish people and slightly fewer Italian and Spanish people. Men were overrepresented in this group (60 %). Compared with other groups, seniors reported a low level of nutritional concern but had an interest in purchasing healthy food products (Figure 2). They were generally less confident about identifying whether a component is a fibre or not, but they recognised the health benefits of DF more often than other groups (see online supplementary material, Supplemental Figure S3a and S3b). People in this group were also the most resistant to incentives for increasing DF intake: they most frequently chose the response ‘none of these would encourage me to eat more high-fibre foods’.

##### ‘Blue collar’ group

**Figure 3.**
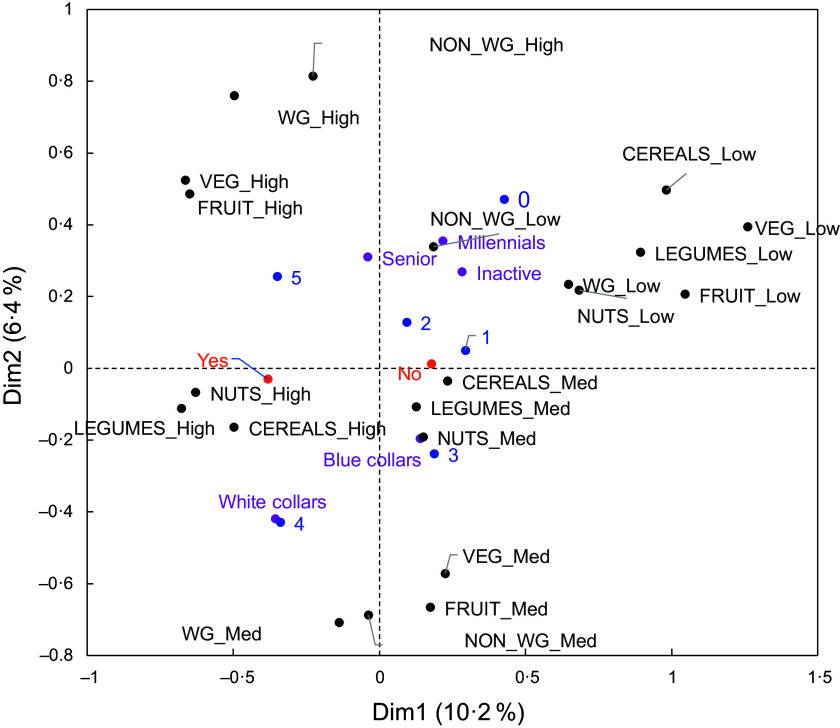
Multiple correspondence analysis using the socio-demographic groups (in purple), degree of nutritional concern (score range: 0–5; in blue), health as a major criterion in food purchases (yes/no; in orange) and relative intake of fibre-rich foods (low/medium/high; in black). The horizontal axis distinguishes between (1) respondents who indicated that they consumed enough dietary fibre and that their intake of fibre-rich foods was high and (2) respondents who indicated that they did not know their dietary fibre intake or that they did not consume enough dietary fibre and who also indicated that their intake of fibre-rich foods was low. There is greater proximity between respondents who both had a high level of nutritional concern and reported that purchasing healthy foods was a major criterion in their shopping choices and respondents who reported that they consumed enough dietary fibre and that their intake of fibre-rich foods was high.

**Figure 4.**
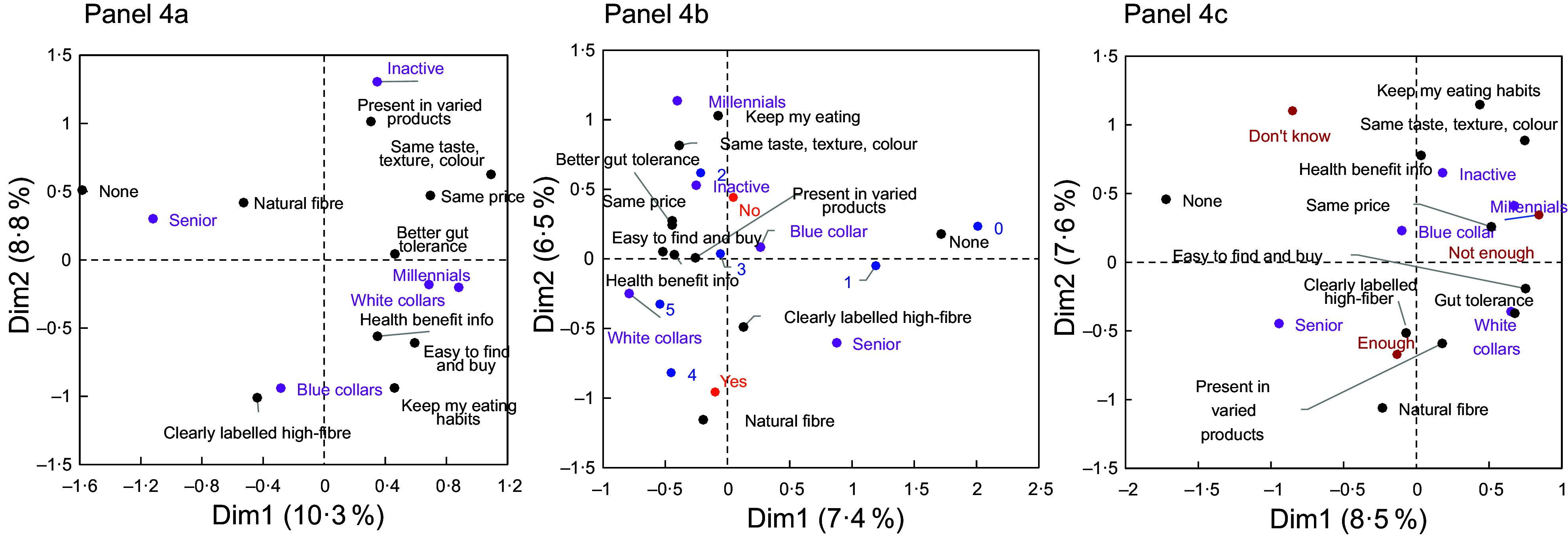
Multiple correspondence analyses using different combinations of variables. Panel 4a: Socio-demographic groups (in purple) and fibre-intake incentives (in black). The horizontal axis distinguishes between individuals who responded ‘none’ and individuals who gave all the other responses to the incentives question, and it also distinguishes between the ‘senior’ group and all the other groups. Panel 4b: Socio-demographic groups (in purple), fibre-intake incentives (in black) and health as a major criterion in food purchases (in orange). The horizontal axis clarifies which respondents had a low level of nutritional concern (score: 0–1), who occur close to respondents who were not interested in any of the proposed incentives (response: ‘none’). The vertical axis distinguishes among respondents who were interested in particular incentives; notably individuals interested in stability (no change in eating habits, maintenance of food sensory characteristics) occurred closer to individuals for whom health was not a major criterion in food purchases. Panel 4c: Socio-demographic groups (in purple), perceived fibre intake (enough/not enough/do not know; in red) and top-ranked incentive (in black). The vertical axis distinguishes the following: respondents who reported eating enough fibre preferred the incentives related to clear labelling, the availability of a wider range of fibre-containing foods and information about naturally occurring fibre; respondents who reported not eating enough fibre occurred close to respondents who mentioned price as a potential incentive; respondents who did not know whether their fibre intake was sufficient appear more prone to have responded ‘none’ relative to the incentives.

This group contained 24 % of all respondents. Most often, they were 45–64 years old (58 %) and lived in households without children (77 %). Many had an intermediate level of income (66 %), and they tended to be actively working: they were often manual labourers (24 %) or employees (39 %). Germany had greater representation in this group, especially compared to Italy and Spain. Also, there were many more men (61 %) than women (39 %). With regard to DF-related questions, their responses were generally reflective of those for the whole study population (this group occurs near the intersection of the main MCA axes (Figures 2 and 3, and see online supplementary material, Supplemental Figure S3)), but they more frequently responded that they would be more likely to eat more high-fibre foods if they could keep their current eating habits and foods were more clearly labelled (Figure 4a).

##### ‘White collar’ group

This group contained a quarter of all respondents (25·5 %). The vast majority (93 %) were young to middle-aged adults (i.e. between 25 and 54 years of age); 87 % lived in households with children. Of the people in this group, only 2 % had a low level of education, while 57 % were highly educated. They most often worked in managerial positions of an intermediate level (20 %) or a high level (31 %), and their income level was either high (46 %) or intermediate (49 %). There was fairly equal representation of men (48 %) and women (52 %). They reported a high level of nutritional concern, and purchasing healthy food products was a major criterion in their shopping decisions. They more frequently responded that they had a medium to high intake of fibre-rich foods and that they consumed enough DF (Figures 2 and 3).

##### ‘Inactive’ group

This group contained 16·7 % of respondents. A large percentage of the people in this group (68 %) were unemployed but not retired, and an even larger percentage were women (71·5 %). They reported a limited level of nutritional concern, and purchasing healthy foods was not a major criterion in their shopping decisions (Figure 2). Their knowledge about DF was similar to the one of other groups. Regarding the incentives, and compared with the whole population, they more frequently expressed a preference for a wider range of high-fibre foods but less frequently requested a clearer product labelling.

##### ‘Millennial’ group

This group contained 9·1 % of respondents. They were mostly younger (73 % were less than 34 years of age), women (78 %) and students (56 %) with a low level of income (76 %). When making shopping decisions, people in this group were less interested than others in purchasing healthy foods. They more frequently reported that their intake of fibre-rich foods was low and that they did not consume enough DF (Figure 2).

#### Relationship between response clusters and socio-demographic groups

**Figure 5.**
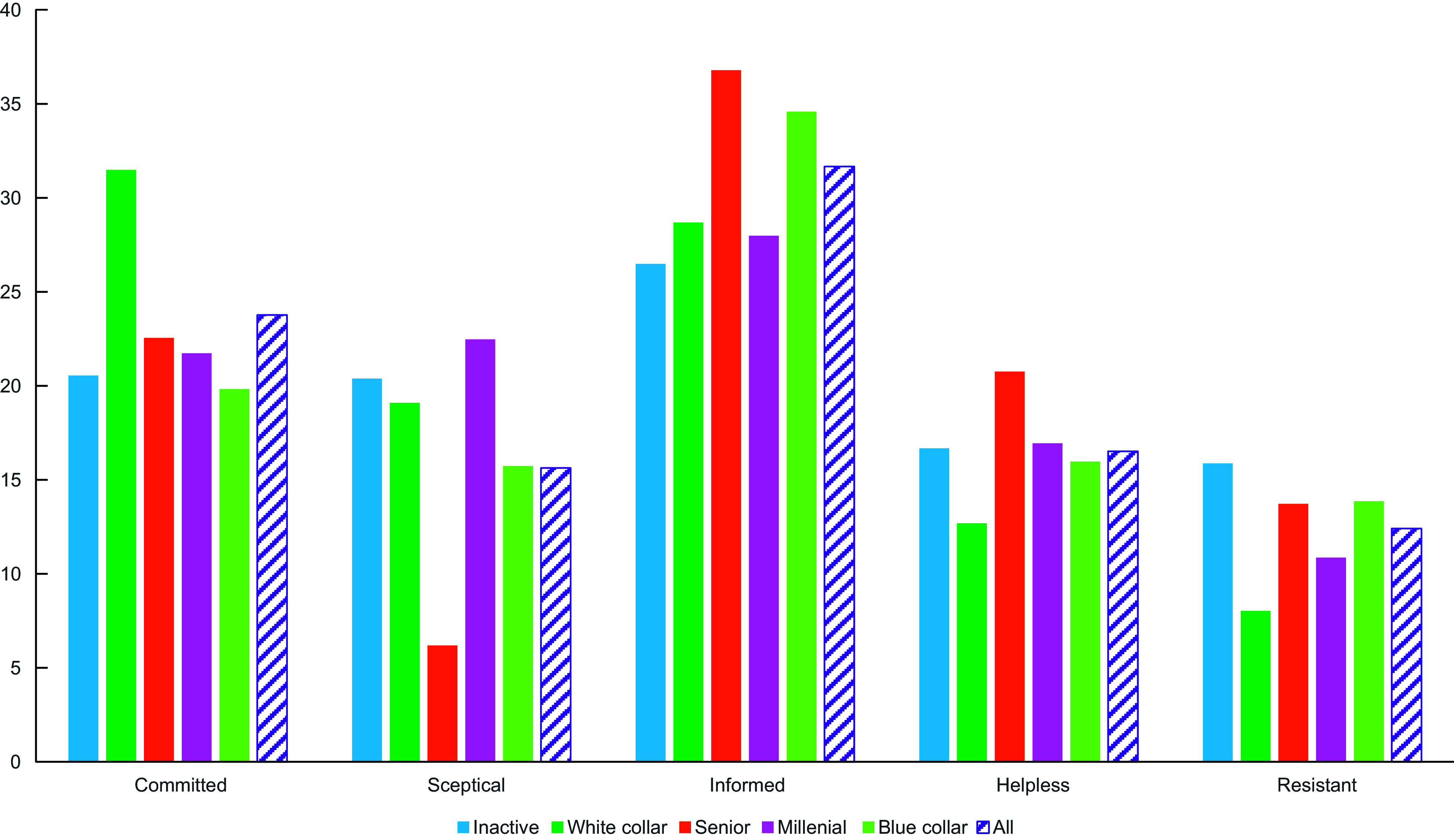
Percent representation of the different response clusters within each socio-demographic group and the whole study population.

The pattern of response clusters within the socio-demographic groups was also examined. Overall, people with different socio-demographic profiles were present in each response cluster, which means that knowledge, perceived intake and attitudes related to DF were not highly dependent on socio-demographic characteristics (Figure 5). That said, there were particular patterns of response profiles within the socio-demographic groups. For example, there was more ‘committed consumers’ in the ‘white collar’ group, compared with the whole study population (+8 percentage points *v*. the proportion of ‘committed consumers’ in the whole population) but less in the ‘blue collar’ group (–5 percentage points). ‘Helpless’ and ‘resistant’ consumers occurred at relatively lower levels in the ‘white collar’ group (–4 points each) compared with the whole study population. People in the ‘senior’ group occurred at relatively higher levels within the clusters of ‘informed’ consumers (+5 points) and ‘helpless’ consumers’ (+4 points). The percentage of ‘sceptical’ was relatively higher in the ‘inactive’ group (+5 points) and in the ‘millennial’ group (+7 points) but relatively lower in the ‘senior’ group (–9 points).

## Discussion

The results of our survey show that the study population generally had a basic understanding of DF: most could identify high-fibre food types, and three out of four respondents were able to identify bran as a DF. That said, there was some confusion around other fibre types. Respondents also associated a high intake of DF with improved intestinal transit, as well as with other proposed health benefits, which were often viewed quite positively. Some respondents even associated DF intake with outlier health benefits, such as increased muscle strength or reduced risks of cognitive decline. This favourable image of fibre and its association with several, if not many, health benefits has been observed in other studies focused on Portuguese people^(19)^, Croatian people^(20)^ and highly educated Saudi Arabians^(21)^. Interestingly, in the latter study, around 70 % of the population strongly associated high DF intake with having a healthier body weight or lower levels of cholesterol, but only 16 % of the population named the relationship with intestinal transit. In contrast, in a study conducted in the US, around 70 % of the population^(22)^ associated DF intake with intestinal transit. These results suggest there could be cultural differences in the perception of these health benefits. In our study of seven European countries, no such differences were observed (see online supplementary material, Supplemental Tables S3–S2).

Here, half of the respondents believed they consumed enough DF, and they usually declared a high frequency of intake of fibre-rich foods. Information about recommendation were not provided, so the spontaneous perception of respondents is recorded. It is worth noting that there is a large discrepancy between such perceptions and actual intake, as seen in dietary surveys. For example, 42·8 % of Spanish respondents indicated that they consumed enough DF, which would be 30 g per day according to nutritional recommendations. However, a nation-wide dietary survey conducted in Spain found that the median daily DF intake for adults was 11·63 g^(23)^, suggesting that the percentage of the population actually consuming sufficient amounts of DF is much lower than 42·8 %. The situation was likely similar for French respondents: 43·5 % indicated that they consumed enough DF, but actual figures show that only 12·6 % of the French population actually does so^(24)^. Among the French respondents, 42·7 % and 46·3 % reported that they had a high daily consumption of fruits and vegetables, respectively, and these figures fit with actual observations (41·7 % for both). At the same time, 45·6 % and 27·5 % indicated that they frequently consumed legumes and whole-grain foods, respectively, figures that are at odds with the results of a dietary survey indicating that over 60 % of French adults do not consume these food products at all^(24)^. Similarly, 45 % of British respondents expressed that they consumed sufficient amounts of DF, when dietary surveys have indicated that only 9·6 % of them actually do^(25)^. Among British respondents, 41·1 % and 45·1 % reported high levels of fruit and vegetable consumption, respectively, these figures are closer to actual observations (around 32 %) but are still overestimates. Previous research has shown that consumers tend to have misperceptions regarding actual food intake: while consumption of junk food or sweetened beverages is often underestimated^(26)^, consumption of fruits and vegetables is often overestimated^(27)^, suggesting a desirability bias that leads respondents to overestimate their intake of healthier foods. This pattern has also been seen in other countries. For example, in Brazil, more than 78 % of a representative adult sample reported regularly consuming fibre^(28)^, which was far from the 13·1 % actually seen to achieve recommended levels of intake in another study^(29)^.

When respondents were asked to rank incentives for increasing their consumption of high-fibre foods, they expressed a clear preference for better labelling and more information about the health benefits. There was also interest in having easier access to a wider range of fibre-rich foods as well as in having information about the presence of naturally occurring fibres. To our knowledge, research on fibre-intake incentives has focused on children and highlighted the role of parents and schools^(30,31)^. The study in Saudi Arabia noted that adults attributed their low consumption of DF to its limited availability and high cost, as well as to the unpleasant taste of fibre-rich foods^(21)^. All three concerns emerged in this study as well.

The clustering analyses captured some of the complexity in the data because they could combine several socio-demographic characteristics (e.g. age, gender, income, employment and country) with responses related to respondent knowledge, perceived intake and attitudes related to DF.

Several response profiles emerged and offered clues about people’s behaviours around food, nutrition, health, in general, and fibre in particular (Figure 1 and see online supplementary material, Supplemental Table S6). The specificities of the different response clusters could translate into recommendations for targeted communication. For example, committed consumers appeared to be ‘pro-fibre’: people in this cluster were relatively more interested in nutrition and health, they believed in the health benefits of fibre and they reported that they consumed high intakes of fibre and fibre-rich foods. Simultaneously, they remained open to increasing their intake further and expressed an interest in a wide range of incentives. They could be a group that is relatively easy to reach with targeted communication. On the opposite end were the ‘resistant’ consumers, who are likely to be more difficult to reach. People in this cluster were relatively less interested in nutrition and health, answered ‘I do not know’ to all the knowledge-related questions, and indicated that none of the incentives would increase their consumption of high-fibre foods. Between these two clusters were the ‘informed’ consumers, who could potentially be targeted using the incentives that appealed the most to them: more informative food labels, a wider range of high-fibre foods available at the same price as their low-fibre equivalents.

The relationships between these response clusters and the socio-demographic groups were also examined to clarify people’s behavioural patterns. It was clear that certain response profiles were more strongly associated with certain socio-demographic groups. For example, ‘committed consumers’ were relatively more common in the ‘white collar’ group, which fits with several past studies in which people with a higher level of education (more common in the ‘white collar’ group in this study) were better informed about DF and reported greater levels of DF consumption^(20,32,33)^. In contrast, in the ‘senior’ group, which contained mostly older individuals, ‘helpless’ consumers were common. The latter were characterised by their lower levels of reported DF intake. This pattern was the opposite of that seen in a study in Brazil, where older people had higher levels of reported DF intake^(28)^.

However, the relatively greater or lesser representation of the response clusters within the socio-demographic groups never exceeded 10 % and socio-demographics explained only part of the observed specificities. This finding does not mean that socio-demographics were not important: in this study, as in many others, factors such as age, income level and educational level influenced people’s knowledge, perceived intake and attitudes related to DF. However, socio-demographic factors likely interact in somewhat compensatory ways. For example, a young educated woman with a lower income would be likely to have an average understanding of DF because, as educated women tended to be more knowledgeable, young people with lower incomes tended to be less knowledgeable. Thus, while approaches based on a single characteristic may be relevant and easier^(34)^, those combining several dimensions may be more successful.

The ultimate goal of public health efforts is to change consumer behaviours, such as increasing DF consumption. Numerous theories and models for bringing about behavioural changes have been developed, and the resulting recommendations can be applied either via individual counselling or population-wide campaigns. More research has been dedicated to the former^(35)^ because monitoring the outcomes of large-scale actions is difficult.

Among the behavioural change models is COM B, a framework based on three essential conditions: Capability, Opportunity and Motivation^(36)^. This model provides a way to design interventions aimed at behavioural changes. The starting point is to consider the behavioural target (in this study, increasing DF intake) and then to define which system components to change and how. The differences highlighted between the clusters identified in our analysis may help to adapt these changes in system components to the characteristics and expectations of consumers.

In the COM B framework, capability means having the necessary knowledge and skills to support change. Indeed, numerous studies have found that higher levels of knowledge are associated with healthier food choices: among US college students, having greater nutritional knowledge increased the likelihood of meeting dietary guidelines regarding the intake of fruits, dairy products, protein and whole grains^(37)^. Similarly, several studies have confirmed that an interest in health and nutrition is associated with more positive dietary attitudes and choices^(38,39)^. Here, the respondents that were the best informed about DF (the ‘committed consumers’) are also those who more often claim high intakes of DF and fibre-rich foods. Thus, we must continue to disseminate information and educate consumers: our data suggest that such actions might be especially, but not exclusively, useful when targeting groups such as ‘helpless’ who seem to lack information, while remaining receptive to receiving it. Following a social marketing campaign in France, it was found that individuals with lower levels of education had become better informed about the fibre content of whole-grain foods^(40)^. A study in the US assessed the efficacy of general (i.e. the food pyramid) *v*. tailored messaging when communicating information about fibre and fibre-rich foods^(41)^. Tailored messaging, meaning messaging that draws upon current beliefs, knowledge structures, attitudes, influences and behaviours, was found to be more effective in increasing food-related knowledge and promoting favourable dietary changes. Our results could help inform efforts to design tailored messaging and actions that target a specific cluster, e.g. using their preferred media, featuring characters they can easily identified as close to them and using familiar language and arguments

The second dimension of the COM B framework is opportunity. In this context, opportunities are all the factors that are external to the individual. To our knowledge, our survey is the first to show that most respondents felt that increasing the range and availability of high-fibre foods was an attractive incentive, or an opportunity, for increasing DF intake. People often dislike high-fibre foods because of their sensory characteristics and such is also true for whole-grain foods^(42,43)^ as well as for other fibre-rich foods^(44,45)^. The food industry must now do its part to produce more whole-grain and fibre-rich foods that better fit consumer expectations for convenient and natural products. Such could be achieved by using more fibre-rich ingredients in recipes, including whole grains, legumes, nuts and seeds. There is also the possibility of utilising innovative natural ingredients, such as high-amylose maize, green banana flour or recently developed wheat white flours that are especially rich in resistant starch^(46,47)^. Such ingredients may favour a ‘health by stealth’ approach, by increasing the fibre content of breads, pasta or doughs, without the consumer noticing it; indeed, a trained sensory panel reported that despite delivering three times more fibre, pasta made with high amylose wheat had no discernible differences in flavour, aroma or texture when compared with standard pasta^(48)^. The creation of new food products would represent an opportunity for consumers, allowing them to choose naturally fibre-rich foods that also display their preferred sensory characteristics. Indeed, nudging consumers towards healthier decisions (i.e. ‘making the healthy choice the easy choice’) is aligned with the opportunity dimension of COM B framework. This strategy was represented in two of the incentives preferred by respondents in this study: expanding the range of high-fibre foods and offering high-fibre foods at the prices seen for their low-fibre equivalents. The price of fibre-rich foods should also be considered as it is especially relevant for some clusters, such as the ‘helpless consumers’.

The third component of the COM B framework is motivation, which refers to the cognitive processes that energise and direct behaviour, not just those behind goal establishment and conscious decision-making. Increasing capability and opportunity can help significantly boost motivation. However, motivation may remain hard to find for some people, such as the’ resistant’ consumers cluster in this study. For the other response clusters, our findings provide useful information that can be employed to design tailored messaging, educational tools and targeted product opportunities. Ultimately, our work could guide the development of European policies for improving DF intake.

Although the questionnaire did not directly address the intention or desire to consume more DF, our data may provide some clues regarding the ‘intention-behaviour gap’, i.e. the discrepancy between what one says and what he or she actually does. This gap, often studied in relation to sustainable food consumption, involves both individual factors, referring to a collection of variables internal to the consumer, and situational factors, referring to external barriers^(49)^. In the present study, the variations of patterns of response within the socio-demographic groups suggest that individual factors play a role; intrapsychic factors such as emotions or values and personal norms were, however, not addressed. Knowledge and trust, thought to be key determinants for narrowing the gap^(50)^, indeed appear connected to a more favourable trend towards consuming more DF, such as in the ‘committed’ cluster. To the opposite, respondent belonging to the ‘resistant’ cluster, who said that ‘nothing would lead them to increase their fibre intake’ are also those who display limited DF knowledge. Similarly, respondents from the ‘sceptical’ cluster have no trust in DF health benefits, fear a low digestive tolerance and appear reluctant to change their dietary habits

Our study had some limitations. The major one is that our questionnaire was short and contained only close-ended questions. However, it is easier for respondents to become distracted during long questionnaires, which is why we decided to limit question number. Our approach allowed us to obtain questionnaires from over 7000 respondents. This article presents only part of the data obtained. Indeed, other analyses could be carried out, particularly those focused on patterns within and among countries. Also, this study was carried out 18 months after the COVID-19 pandemic began, and respondents’ health-related concerns may have been shaped by the circumstances^(51)^. This study has also strengths; the main ones were the multiple component analyses and the hierarchical clustering analyses, the large size of the study population, the in-depth characterisation of socio-demographics and its country representativeness. The latter means that our conclusions are valid for the seven countries surveyed, which encompass 68 % of the population of the EU-27 plus the UK. The availability of country-specific data enables additional analyses, which may inform more precisely on a specific country, and could explore cross-cultural differences across these seven European countries.

### Conclusion

This study gathered and analysed original data on consumer knowledge, perceived intake and attitudes related to DF for a large representative sample taken from across Europe. Half of the respondents indicated that they consumed enough DF, and most remained open to further increasing their intake, as they generally had a good understanding about the health benefits of a fibre-rich diet. Consumer traits and expectations varied, and the clustering analyses yielded information that can be used to design target policies, tailored messaging and a range of attractive food products. Five clusters were identified, which may enable the implementation of customised campaigns, but still susceptible to reach significant proportion of the population. Further field research remains however needed to confirm the efficacy of such targeted policies to increase DF intake.

## Supporting information

Azaïs-Braesco et al. supplementary material 1 Azaïs-Braesco et al. supplementary material

Azaïs-Braesco et al. supplementary material 2 Azaïs-Braesco et al. supplementary material

## Supplementary material

For supplementary material accompanying this paper visit https://doi.org/10.1017/S1368980026102298
