# Supplementary material for: Consumers vary in their attitudes and expectations about dietary fibre: analysis of answers to a pan-European online survey
Source: Public Health Nutr. 2026 Mar 25;29(1):e74. doi: 10.1017/S1368980026102298 (PMC13087982; doi:10.1017/S1368980026102298)
Supplement: Azaïs-Braesco et al. supplementary material 1 — Azaïs-Braesco et al. supplementary material [file S1368980026102298sup001.docx]

**Supplementary Material - Figures**

**Figure S1: Clustering based on responses**

***Panel A***: Dendrogram derived from the hierarchical clustering analysis. The horizontal line shows where the dendrogram was cut to obtain 5 clusters.


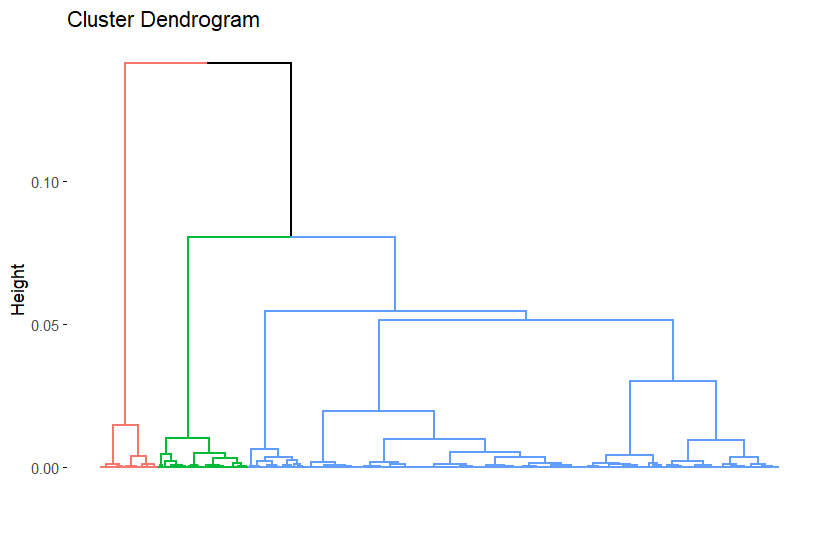


***panel B:*** Representation of respondents along the first three principal components (three dimensions) extracted from the MCA applied to the responses to fibre-related questions. Colours identify the 5 patterns (or clusters) of responses obtained thanks to the hierarchical clustering analysis (1: blue/green: ‘sceptical consumers’; 2: orange: informed consumers; 3: blue: resistant consumers; 4: pink: committed consumers; 5: green: helpless consumers)

B


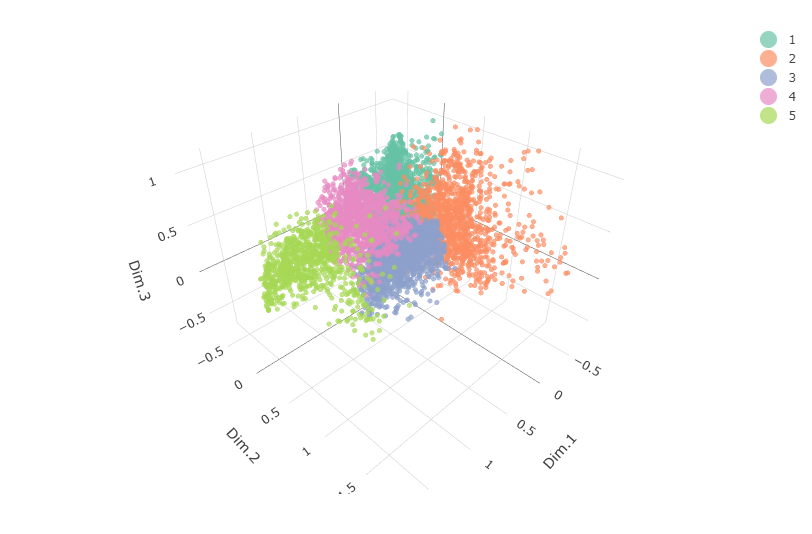


**Figure S2: Clustering based on sociodemographic data**

***Panel A:*** Dendrogram derived from the hierarchical clustering analysis. The horizontal line shows where the dendrogram was cut to obtain 5 clusters.


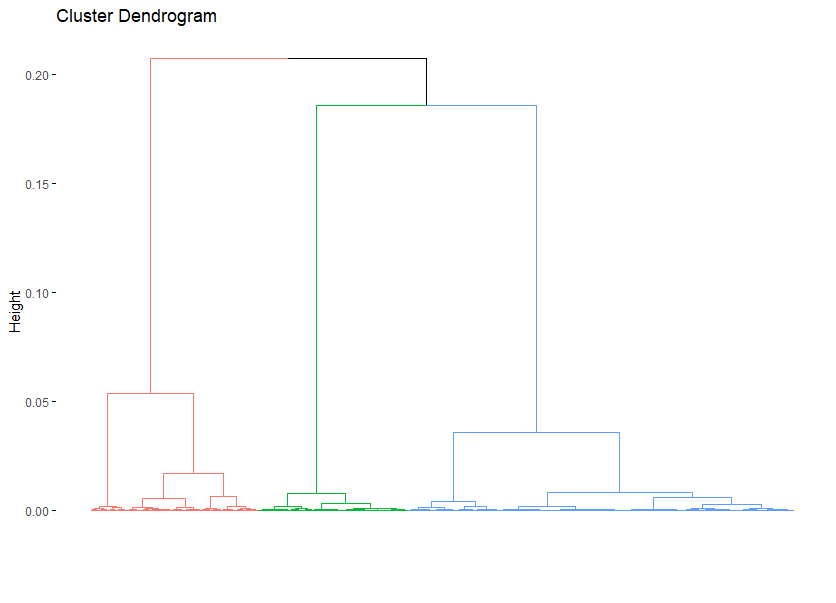


***Panel B:*** Representation of respondents along the first two principal components (two dimensions) extracted from the MCA applied to the sociodemographic data. Colours identify the 5 patterns (or groups) obtained thanks to the hierarchical clustering analysis (1: orange: senior; 2: green: blue collar; 3: blue/green: white collar; 4: blue: inactive; 5: red: millenial).


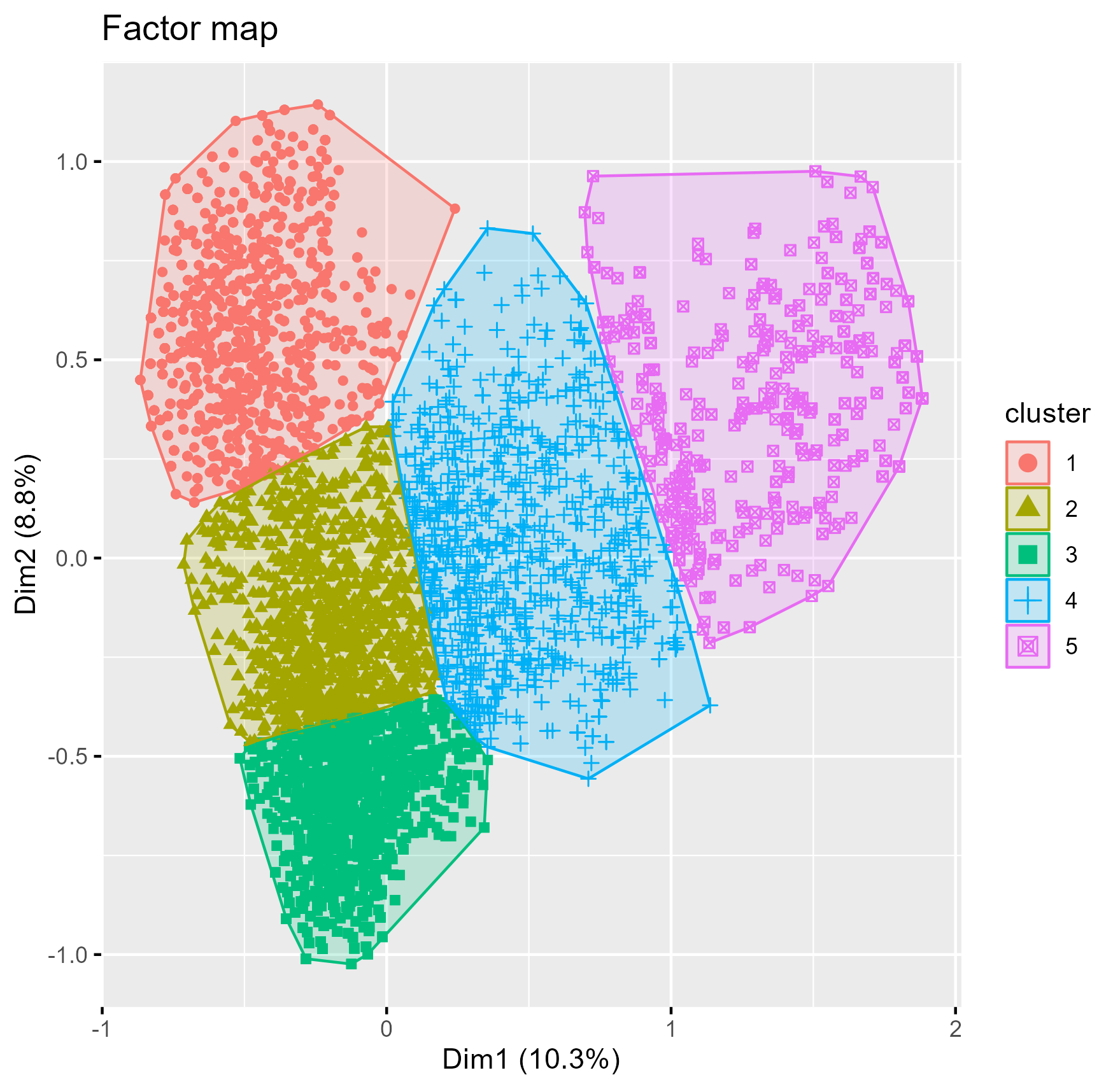


**Figure S3:** MCA using the following variables: sociodemographic groups (purple colour), score of nutritional concern (from 0 to 5; blue colour), health as a major criterion in food purchases (yes/no; grey colour), plus, in orange colour: knowledge of types of fibre (***panel A***), knowledge of health benefits of fibre (***panel B***),.See details of the questionnaire (Supplementary Table S1) for the possible responses.

**Panel A**

**Panel B**
