# Supplementary material for: Consumers vary in their attitudes and expectations about dietary fibre: analysis of answers to a pan-European online survey
Source: Public Health Nutr. 2026 Mar 25;29(1):e74. doi: 10.1017/S1368980026102298 (PMC13087982; doi:10.1017/S1368980026102298)
Supplement: Azaïs-Braesco et al. supplementary material 2 — Azaïs-Braesco et al. supplementary material [file S1368980026102298sup002.docx]

Supplementary material A (Tables):

- Table S1: questionnaire
- Table S2: Socio-demographic characteristics, by country
- Table S3-1: Fibre knowledge. Answers to question “Is this a dietary fibre?’
- Table S3-2: Fibre knowledge. Answers to ‘Is this a health benefit related to a high fibre diet?’
- Table S3-3: Fibre-knowledge. Answers to the question “How do you recognise a high-fibre food?”
- Table S4: Perception of own’s intake of dietary fibre and of frequency of intake of fibre-rich foods.
- Table S5: Incentives to increase intake of dietary fibre. Answer to the question: ‘What would make you eat more of high-fibre foods?’
- Table S6: (see excel file)
- Table S7: Socio-demographic characteristics of the whole sample and for each group of respondents, identified through hierarchical clustering.

**Supplementary material S1: Questionnaire**

Theme 1: nutrition

- Question 1: On a scale from 0 to 5, how concerned are you about nutrition? From 0 = not concerned to 5 = very concerned
- Question 2: When it comes to shopping meals and food, which of the following criteria/on is most important to you? (3 ANSWERS POSSIBLE – First, Second, Third)
  - Presented at random
    - Buying tasty food-products
    - Buying healthy food-products
    - Buying environmentally friendly food-products
    - Satisfying hunger and basic needs
    - Reducing time spent on cooking or eating
    - Spending time with family and friends while having a meal
    - Reducing money spent on food-products
    - Discovering new meals, new tastes

Theme 2: knowledge about dietary fibre

- Question 1: According to you, what types of food contain most dietary fibres? please, rank the listed items below ( first rank, second rank, third rank)
  - Presented at random
    - Legumes (lentils, beans…)
    - Cereals (oats, quinoa, corn…)
    - Cereal based products (bread, pasta, biscuits…)
    - Vegetables (carrots, broccoli…) and fruits (apple, oranges…)
    - Nuts and seeds
    - Dairy products (milk, yogurt, cheese…)
    - Meat, fish, eggs, meat based meat-based products, fish-based products
    - Fats, oils, and butter
    - Potatoes, sweet potatoes
    - Chocolate
    - None
    - I don’t know
- Question 2: According to you, which items from the list below are dietary fibres?
  - For each item, answer by yes, no or “I don’t know”
  - Presented at random
    - Pectin
    - Sugar
    - Vitamins
    - Polyphenols
    - Bran
    - Cellulose
    - Resistant Starch
    - FOS (fructo-oligosaccharides)/GOS (galacto-oligo saccharides)
- Question 3: How do you recognise a high-fibre food?
  - For each item, answer by yes, no or “I don’t know”
  - Presented at random
    - Its colour
    - Its taste
    - Its smell
    - Information on label
    - Its texture
- Question 4: According to you, which items from the list below are the health benefits related to a high-fibre diet?
  - For each item, answer by yes, no or “I don’t know”
  - Presented at random
    - Improvement of intestinal transit
    - Improvement of the immune system
    - Better control of blood sugar
    - Better control of blood cholesterol and cardiovascular health
    - Reduced risks of cognitive decline for the elderly
    - Help keeping a healthy body weight
    - Improvement of muscle strength
    - Positive impact on gut microbiota

Theme 3: perception of own’s dietary intakes

- Question 1: According to you, do you eat (one answer only)
  - Enough dietary fibre
  - Not enough dietary fibre
  - You don’t know
- Question 2: Considering the past 4 weeks did you eat these products
  - - Every day
    - Several times a week
    - Once a week
    - Less often
    - Never
  - the list of products was as follows
    - vegetables
    - fruits
    - legumes (lentils, beans, ...)
    - cereals (rice, quinoa, oat, corn, ...)
    - whole grain cereal products (bread, pasta, biscuits, ...)
    - non whole grain cereal products (bread, pasta, biscuits, ...)
    - nuts and seeds

note: recoding of answers to the above question.

In order to better reflect food-based dietary guidelines which recommend different intakes frequencies for these foods, the answers were recode as follows:

- Fruits & vegetables
  - High frequency: everyday
  - Medium frequency: several times a week
  - Low frequency: all other ones
- Legumes, Cereals (rice, quinoa, …) and nuts and seeds
  - High frequency: every day and several times a week
  - Medium frequency: once a week
  - Low frequency: less often and never
- Whole grain and non-whole grain cereal products (breads, pasta, biscuits)
  - High frequency: everyday
  - Medium frequency: several times a week
  - Low frequency: all other ones

Theme 4: incentives

- Question: What would make you eat more of high-fibre foods? Rank the items listed below (rank 1, rank 2, rank 3)
  - If you had more or better information about their health benefit
  - If you had proof/ evidence that high-fibre foods contain only naturally occurring fibres
  - If high-fibre products were easy to find and easily available in shops
  - If they were more clearly labelled “high-fibre products”?
  - If they would not force me to change my eating habits
  - If I were more tolerant to fibre food
  - If they were present in a wider range of convenience foods (bread, salads, pasta, sandwiches, flours flour, biscuits, ready-to-eat meals, …)
  - If they had the same taste, texture or colour as low-fibre foods
  - If they had the same price as their low-fibre foods
  - None of these would incentivise me to eat more high fibre food
- **Table S2: Socio-demographic characteristics, by country**

| **Country (number of respondents)** | | **All (7427)** | **France (1095)** | **Germany (1076)** | **Italy (1106)** | **Netherlands (1028)** | **Spain (1069)** | **Sweden (1006)** | **UK (1047)** |
| --- | --- | --- | --- | --- | --- | --- | --- | --- | --- |
| **item** | **modality** |  |  |  |  |  |  |  |  |
| AGE | 18-24y | 9,9 | 10,1 | 8,9 | 10,1 | 10,1 | 9,3 | 10,9 | 11,0 |
|  | 25-34y | 15,5 | 16,7 | 14,5 | 14,3 | 13,8 | 16,3 | 16,8 | 16,2 |
|  | 35-44y | 17,2 | 16,4 | 15,4 | 18,7 | 17,2 | 20,8 | 12,6 | 17,0 |
|  | 45-54y | 18,6 | 17,0 | 21,3 | 17,9 | 19,4 | 17,4 | 16,2 | 18,6 |
|  | 55-64y | 15,3 | 16,2 | 16,0 | 14,9 | 16,9 | 13,0 | 15,6 | 15,0 |
|  | >=65y | 23,5 | 23,6 | 23,9 | 24,1 | 22,6 | 23,1 | 27,8 | 22,2 |
| CHILDREN | With | 43,5 | 45,2 | 28,0 | 58,4 | 38,3 | 53,2 | 32,3 | 44,0 |
|  | Without | 56,5 | 54,8 | 72,0 | 41,6 | 61,7 | 46,8 | 67,7 | 56,0 |
| EDUCATION | Low | 15,8 | 8,0 | 23,1 | 11,8 | 29,3 | 19,4 | 11,2 | 12,9 |
|  | Medium | 53,3 | 64,4 | 56,2 | 57,9 | 48,9 | 37,5 | 65,3 | 44,7 |
|  | High | 30,9 | 27,7 | 20,7 | 30,2 | 21,8 | 43,1 | 23,5 | 42,4 |
| GENDER | Man | 48,4 | 45,8 | 49,3 | 46,8 | 48,1 | 49,5 | 50,7 | 50,3 |
|  | Woman | 51,6 | 54,2 | 50,7 | 53,2 | 51,9 | 50,5 | 49,3 | 49,7 |
| INCOME | Low | 26,9 | 28,3 | 26,0 | 25,4 | 30,2 | 25,1 | 32,8 | 27,5 |
|  | Medium | 50,5 | 54,1 | 50,7 | 47,4 | 59,2 | 45,9 | 44,2 | 51,4 |
|  | High | 22,6 | 17,6 | 23,4 | 27,2 | 10,5 | 29,0 | 22,9 | 21,1 |
| JOB SITUATION | At home | 17,0 | 13,1 | 12,6 | 21,3 | 22,4 | 18,0 | 14,1 | 20,9 |
|  | Retired | 25,3 | 30,4 | 27,2 | 22,0 | 21,6 | 22,2 | 32,0 | 22,9 |
|  | Student | 5,2 | 4,2 | 4,3 | 8,1 | 4,8 | 6,0 | 8,1 | 3,9 |
|  | Work | 52,5 | 52,2 | 56,0 | 48,6 | 51,2 | 53,8 | 45,8 | 52,2 |
| Socio Professional Category | Employed | 33,0 | 39,2 | 36,4 | 30,5 | 33,4 | 33,6 | 40,2 | 22,9 |
|  | Inactive | 13,8 | 11,8 | 10,1 | 19,8 | 18,8 | 11,3 | 11,3 | 16,0 |
|  | Management | 18,0 | 12,8 | 22,1 | 18,6 | 16,0 | 15,5 | 17,1 | 19,9 |
|  | Manual worker | 18,3 | 16,4 | 18,5 | 13,7 | 15,2 | 24,0 | 19,4 | 21,1 |
|  | Middle management | 16,9 | 19,8 | 12,9 | 17,4 | 16,5 | 15,6 | 12,0 | 20,2 |

Pvalues for differences across countries are 0.189 for age, 0.33 for gender and are below 0.001 for all the other variables

**Table S3-1: Fibre knowledge. Answers to the question “Is this a dietary fibre?’**

|  | **modality** | **All** | **France** | **Germany** | **Italy** | **Netherlands** | **Spain** | **Sweden** | **UK** |
| --- | --- | --- | --- | --- | --- | --- | --- | --- | --- |
| **Is this a dietary fibre?** | | | | | | | | | |
| Bran | Don't know | 18,6 | 27,3 | 22,7 | 8,5 | 17,8 | 21,5 | 21,1 | 11,6 |
|  | No | 7,6 | 12,4 | 8,2 | 3,4 | 6,8 | 9,0 | 6,1 | 5,4 |
|  | Yes | 73,8 | 60,3 | 69,1 | 88,1 | 75,5 | 69,5 | 72,8 | 83,0 |
| Cellulose | Don't know | 40,2 | 39,2 | 39,3 | 34,3 | 43,7 | 39,4 | 37,4 | 47,8 |
|  | No | 26,2 | 24,8 | 25,8 | 19,8 | 32,5 | 30,6 | 24,6 | 29,3 |
|  | Yes | 33,6 | 35,9 | 34,9 | 45,9 | 23,8 | 30,0 | 38,0 | 22,9 |
| FOS-GOS | Don't know | 55,8 | 55,7 | 53,5 | 49,1 | 65,2 | 57,7 | 52,1 | 61,5 |
|  | No | 28,8 | 26,1 | 35,8 | 30,4 | 22,6 | 24,0 | 35,9 | 25,1 |
|  | Yes | 15,4 | 18,2 | 10,7 | 20,5 | 12,2 | 18,3 | 12,0 | 13,4 |
| Polyphenols | Don't know | 59,6 | 56,6 | 61,2 | 49,5 | 66,6 | 61,0 | 64,1 | 66,0 |
|  | No | 27,1 | 30,1 | 29,3 | 31,9 | 20,9 | 22,8 | 24,4 | 22,0 |
|  | Yes | 13,4 | 13,3 | 9,5 | 18,6 | 12,5 | 16,2 | 11,5 | 12,0 |
| pectin | Don't know | 49,7 | 43,5 | 47,8 | 46,3 | 58,5 | 55,5 | 46,1 | 55,4 |
|  | No | 29,6 | 28,9 | 31,6 | 31,5 | 28,0 | 22,8 | 41,9 | 29,2 |
|  | Yes | 20,8 | 27,6 | 20,6 | 22,2 | 13,5 | 21,7 | 12,0 | 15,4 |
| Resistant Starch | Don't know | 46,0 | 44,9 | 47,7 | 41,2 | 40,4 | 45,5 | 47,4 | 51,1 |
|  | No | 26,4 | 24,7 | 26,6 | 28,3 | 25,5 | 29,7 | 33,9 | 22,9 |
|  | Yes | 27,6 | 30,4 | 25,7 | 30,4 | 34,1 | 24,9 | 18,7 | 26,0 |
| Sugar | Don't know | 23,9 | 26,3 | 23,9 | 25,5 | 20,2 | 20,9 | 22,1 | 23,3 |
|  | No | 63,0 | 59,0 | 59,1 | 62,2 | 64,7 | 69,9 | 68,9 | 66,2 |
|  | Yes | 13,2 | 14,8 | 17,0 | 12,3 | 15,1 | 9,2 | 9,0 | 10,5 |
| Vitamins | Don't know | 27,7 | 30,1 | 24,9 | 26,5 | 23,8 | 28,2 | 26,6 | 30,6 |
|  | No | 46,2 | 38,0 | 59,6 | 48,9 | 46,8 | 40,4 | 54,0 | 38,0 |
|  | Yes | 26,1 | 32,0 | 15,5 | 24,6 | 29,5 | 31,4 | 19,3 | 31,4 |

**Table S3-2: Fibre knowledge. Answers to the question ‘Is this a health benefit related to a high fibre diet?’**

|  | | **modality** | | **All** | | **France** | | **Germany** | | **Italy** | | **Netherlands** | | | **Spain** | | **Sweden** | | **UK** |
| --- | --- | --- | --- | --- | --- | --- | --- | --- | --- | --- | --- | --- | --- | --- | --- | --- | --- | --- | --- |
|  | | | | | | | | | | | | | | | | | | |  |
| **Is this a benefit associated with a high fibre diet?** | | | | | | | | | | | | | | | | | | |  |
| Improvement of blood cholesterol | | Don't know | | 26,9 | | 28,6 | | 29,4 | | 20,9 | | 29,5 | 23,1 | | 30,8 | | 28,6 | |  |
|  |  | No | | 11,2 | | 12,2 | | 15,8 | | 7,5 | | 13,5 | 7,2 | | 10,7 | | 10,2 | |  |
|  |  | Yes | | 61,9 | | 59,2 | | 54,8 | | 71,6 | | 56,9 | 69,7 | | 58,5 | | 61,2 | |  |
| Improvement of blood sugar | | Don't know | | 29,3 | | 31,5 | | 29,7 | | 25,5 | | 27,4 | 29,1 | | 25,1 | | 31,4 | |  |
|  |  | No | | 13,7 | | 15,4 | | 15,6 | | 12,0 | | 12,8 | 13,0 | | 10,4 | | 12,4 | |  |
|  |  | Yes | | 57,0 | | 53,1 | | 54,7 | | 62,6 | | 59,8 | 57,9 | | 64,5 | | 56,2 | |  |
| Improvement of gut microbiota | | Don't know | | 23,6 | | 15,8 | | 44,5 | | 12,1 | | 14,9 | 14,7 | | 12,2 | | 25,9 | |  |
|  |  | No | | 8,3 | | 8,5 | | 12,2 | | 5,4 | | 5,5 | 7,3 | | 6,0 | | 7,8 | |  |
|  |  | Yes | | 68,1 | | 75,7 | | 43,3 | | 82,5 | | 79,6 | 78,0 | | 81,7 | | 66,3 | |  |
| Improvement of the immune system | | Don't know | | 29,0 | | 30,5 | | 28,6 | | 27,9 | | 25,7 | 27,3 | | 32,0 | | 30,4 | |  |
|  |  | No | | 15,2 | | 15,5 | | 18,5 | | 16,2 | | 13,5 | 11,6 | | 17,4 | | 12,7 | |  |
|  |  | Yes | | 55,8 | | 54,0 | | 53,0 | | 55,9 | | 60,8 | 61,1 | | 50,6 | | 56,9 | |  |
| Improvement of muscle strength | | Don't know | | 34,0 | | 32,9 | | 34,6 | | 33,3 | | 29,9 | 32,4 | | 34,8 | | 37,1 | |  |
|  |  | No | | 22,8 | | 20,9 | | 29,2 | | 21,4 | | 21,2 | 18,4 | | 26,0 | | 21,1 | |  |
|  |  | Yes | | 43,2 | | 46,2 | | 36,2 | | 45,3 | | 48,9 | 49,2 | | 39,2 | | 41,8 | |  |
| Improvement of intestinal transit | | Don't know | | 13,4 | | 13,8 | | 14,1 | | 8,7 | | 10,3 | 9,0 | | 10,4 | | 20,6 | |  |
|  |  | No | | 6,6 | | 7,7 | | 8,3 | | 5,3 | | 5,0 | 4,3 | | 5,0 | | 7,0 | |  |
|  |  | Yes | | 80,0 | | 78,5 | | 77,6 | | 86,0 | | 84,7 | 86,7 | | 84,6 | | 72,4 | |  |
| help in keeping a healthy body weight | | Don't know | | 19,9 | | 24,0 | | 23,3 | | 14,4 | | 19,4 | 13,7 | | 20,8 | | 20,8 | |  |
|  |  | No | | 10,8 | | 11,3 | | 14,8 | | 9,6 | | 10,2 | 7,3 | | 11,5 | | 9,0 | |  |
|  |  | Yes | | 69,3 | | 64,7 | | 61,8 | | 76,0 | | 70,4 | 79,0 | | 67,7 | | 70,2 | |  |
| reduction of the risk of cognitive decline | | Don't know | | 43,0 | | 41,1 | | 47,4 | | 41,7 | | 40,9 | 38,7 | | 48,2 | | 43,5 | |  |
|  |  | No | | 16,2 | | 15,7 | | 20,7 | | 15,6 | | 15,8 | 12,1 | | 15,8 | | 14,6 | |  |
|  |  | Yes | | 40,8 | | 43,3 | | 31,9 | | 42,6 | | 43,3 | 49,2 | | 36,0 | | 42,0 | |  |

**Table S3-3: Fibre knowledge. Answers to the question “How do you recognise a high-fibre food?”**

|  | **modality** | **All** | **France** | **Germany** | **Italy** | **Netherlands** | **Spain** | **Sweden** | **UK** |
| --- | --- | --- | --- | --- | --- | --- | --- | --- | --- |
| by its color | Don't know | 26,9 | 25,2 | 28,7 | 27,3 | 29,2 | 24,2 | 28,7 | 27,1 |
|  | No | 52,7 | 53,0 | 61,7 | 42,4 | 50,3 | 53,1 | 50,8 | 51,3 |
|  | Yes | 20,3 | 21,8 | 9,5 | 30,2 | 20,5 | 22,7 | 20,5 | 21,6 |
| by the information on label | Don't know | 12,1 | 13,8 | 17,2 | 8,1 | 12,1 | 8,3 | 12,8 | 10,0 |
|  | No | 7,6 | 11,5 | 8,9 | 4,3 | 5,1 | 6,0 | 4,8 | 7,2 |
|  | Yes | 80,4 | 74,7 | 73,9 | 87,7 | 82,8 | 85,7 | 82,3 | 82,8 |
| by its smell | Don't know | 27,2 | 24,4 | 28,5 | 28,4 | 30,4 | 24,0 | 30,7 | 28,1 |
|  | No | 58,6 | 58,4 | 62,2 | 55,8 | 52,5 | 60,3 | 56,8 | 57,5 |
|  | Yes | 14,2 | 17,2 | 9,2 | 15,8 | 17,1 | 15,7 | 12,4 | 14,4 |
| by its taste | Don't know | 25,2 | 23,6 | 28,0 | 26,0 | 26,3 | 20,7 | 26,2 | 25,6 |
|  | No | 44,0 | 43,6 | 51,4 | 38,3 | 40,1 | 45,6 | 39,2 | 41,0 |
|  | Yes | 30,7 | 32,8 | 20,6 | 35,7 | 33,5 | 33,7 | 34,6 | 33,4 |
| by its texture | Don't know | 25,3 | 20,7 | 30,5 | 28,7 | 24,7 | 21,1 | 26,6 | 23,3 |
|  | No | 29,9 | 26,1 | 32,9 | 36,2 | 18,8 | 33,3 | 17,9 | 26,9 |
|  | Yes | 44,8 | 53,2 | 36,6 | 35,2 | 56,5 | 45,5 | 55,5 | 49,8 |

**Table S4: Perception of own’s intake of dietary fibre and of frequency of intake of fibre-rich foods.**

| **question** | **modality** | **All** | **France** | **Germany** | **Italy** | **Netherlands** | **Spain** | **Sweden** | **UK** |
| --- | --- | --- | --- | --- | --- | --- | --- | --- | --- |
| do you eat enough fibre? | I don't know | 21,1 | 22,4 | 22,5 | 17,1 | 17,8 | 23,2 | 20,8 | 21,2 |
|  | not enough | 29,2 | 34,1 | 29,3 | 18,0 | 14,2 | 34,0 | 36,5 | 33,8 |
|  | enough | 49,7 | 43,5 | 48,2 | 65,0 | 68,0 | 42,8 | 42,7 | 45,0 |
| how often do you eat cereals (rice, quinoa, oat, corn,..)? | low frequency | 19,1 | 20,2 | 23,0 | 17,8 | 19,8 | 11,7 | 17,1 | 19,4 |
|  | medium frequency | 29,5 | 33,2 | 32,8 | 34,3 | 30,1 | 29,4 | 24,9 | 18,0 |
|  | high frequency | 51,4 | 46,5 | 44,2 | 48,0 | 50,1 | 58,9 | 58,0 | 62,6 |
| how often do you eat fruits? | low frequency | 20,2 | 17,2 | 24,5 | 15,0 | 17,1 | 16,8 | 33,5 | 23,5 |
|  | medium frequency | 37,3 | 40,1 | 45,5 | 31,0 | 31,5 | 32,4 | 34,3 | 35,3 |
|  | high frequency | 42,5 | 42,7 | 30,0 | 53,9 | 51,5 | 50,7 | 32,2 | 41,1 |
| how often do you eat legumes (lentils, beans,..) | low frequency | 25,3 | 20,6 | 34,2 | 16,7 | 25,4 | 10,5 | 45,9 | 34,1 |
|  | medium frequency | 34,8 | 33,8 | 39,2 | 35,6 | 38,9 | 35,5 | 27,3 | 29,2 |
|  | high frequency | 39,9 | 45,6 | 26,6 | 47,7 | 35,7 | 54,0 | 26,8 | 36,7 |
| how often do you eat whole-grain cereal-based products (bread, pasta, biscuits,…)? | low frequency | 35,4 | 27,2 | 43,5 | 28,0 | 44,2 | 34,4 | 39,3 | 38,2 |
|  | medium frequency | 43,0 | 45,3 | 43,3 | 41,7 | 37,6 | 42,0 | 39,5 | 44,1 |
|  | high frequency | 21,6 | 27,5 | 13,3 | 30,3 | 18,1 | 23,6 | 21,2 | 17,7 |
| how often do you eat nuts and seeds? | low frequency | 33,8 | 42,1 | 33,2 | 27,3 | 27,0 | 20,6 | 39,4 | 42,3 |
|  | medium frequency | 23,8 | 25,1 | 25,8 | 24,1 | 22,7 | 22,2 | 27,2 | 20,5 |
|  | high frequency | 42,4 | 32,8 | 40,9 | 48,5 | 50,3 | 57,2 | 33,4 | 37,2 |
| how often do you eat vegetables? | low frequency | 13,1 | 11,1 | 14,4 | 11,6 | 6,1 | 17,7 | 16,7 | 13,0 |
|  | medium frequency | 46,3 | 46,3 | 52,8 | 39,9 | 38,9 | 53,7 | 38,0 | 41,9 |
|  | high frequency | 40,6 | 42,5 | 32,8 | 48,5 | 55,0 | 28,7 | 45,3 | 45,1 |
| how often do you eat non whole-grain cereal-based products (bread, pasta, biscuits,…)? | low frequency | 30,6 | 26,2 | 35,4 | 32,6 | 17,2 | 29,9 | 29,0 | 31,7 |
|  | medium frequency | 40,8 | 41,6 | 42,8 | 37,8 | 33,6 | 42,6 | 41,0 | 40,6 |
|  | high frequency | 28,6 | 32,1 | 21,8 | 29,6 | 49,2 | 27,5 | 30,0 | 27,8 |

**Table S5: Incentives to increase intake of dietary fibre. Answer to the question: ‘What would make you eat more of high-fibre foods?’**

|  | **modalite** | **All** | **France** | **Germany** | **Italy** | **Netherlands** | **Spain** | **Sweden** | **UK** |
| --- | --- | --- | --- | --- | --- | --- | --- | --- | --- |
| first rank | Better gut tolerance | 4,7 | 4,8 | 3,9 | 5,2 | 3,7 | 3,7 | 8,5 | 5,4 |
|  | Clearly labelled high-fibre | 17,0 | 17,3 | 19,2 | 15,9 | 16,3 | 14,4 | 14,3 | 17,5 |
|  | Easy to find and buy | 8,1 | 8,2 | 6,9 | 6,3 | 7,0 | 10,4 | 9,8 | 9,4 |
|  | Health benefit info | 10,6 | 13,6 | 9,7 | 10,1 | 7,5 | 12,2 | 7,7 | 9,6 |
|  | Keep my eating habits | 6,2 | 6,9 | 6,2 | 6,0 | 4,8 | 7,6 | 5,5 | 5,2 |
|  | Natural fibre | 9,5 | 11,7 | 8,0 | 12,4 | 6,2 | 11,2 | 6,5 | 6,4 |
|  | None | 12,7 | 11,4 | 18,6 | 8,4 | 19,1 | 6,8 | 14,1 | 12,9 |
|  | Present in varied products | 13,7 | 11,9 | 12,2 | 16,9 | 16,1 | 12,9 | 11,8 | 14,6 |
|  | Same price | 10,5 | 9,2 | 9,1 | 11,7 | 12,4 | 12,5 | 13,2 | 10,4 |
|  | Same taste, texture, colour | 7,0 | 4,9 | 6,2 | 7,1 | 6,9 | 8,4 | 8,6 | 8,6 |
| second rank | Better gut tolerance | 3,6 | 4,1 | 4,4 | 2,0 | 2,9 | 3,5 | 4,8 | 3,8 |
|  | Clearly labelled high-fibre | 14,2 | 14,0 | 13,9 | 15,6 | 12,9 | 14,4 | 12,6 | 13,7 |
|  | Easy to find and buy | 10,1 | 8,7 | 10,8 | 8,7 | 12,5 | 12,5 | 11,2 | 9,5 |
|  | Health benefit info | 10,0 | 12,2 | 8,4 | 10,8 | 8,0 | 9,2 | 9,4 | 10,1 |
|  | Keep my eating habits | 7,4 | 8,6 | 7,5 | 7,6 | 5,6 | 7,8 | 5,9 | 6,3 |
|  | Natural fibre | 9,5 | 11,7 | 8,5 | 12,7 | 6,4 | 10,4 | 6,3 | 6,4 |
|  | Nothing else | 14,7 | 13,6 | 20,5 | 9,4 | 22,8 | 8,2 | 16,4 | 15,6 |
|  | Present in varied products | 12,4 | 10,6 | 11,1 | 14,0 | 11,1 | 12,6 | 13,8 | 14,2 |
|  | Same price | 10,2 | 10,8 | 8,1 | 10,9 | 9,9 | 10,9 | 10,8 | 11,3 |
|  | Same taste, texture, colour | 7,9 | 5,7 | 6,9 | 8,3 | 8,0 | 10,5 | 8,8 | 9,0 |
| third rank | Better gut tolerance | 3,9 | 4,6 | 3,7 | 3,6 | 3,0 | 3,7 | 3,9 | 4,2 |
|  | Clearly labelled high-fibre | 11,3 | 11,5 | 10,0 | 13,9 | 10,5 | 12,7 | 11,9 | 9,6 |
|  | Easy to find and buy | 10,9 | 10,1 | 11,5 | 8,9 | 10,8 | 11,9 | 14,0 | 11,7 |
|  | Health benefit info | 10,0 | 12,2 | 8,4 | 11,6 | 8,4 | 9,7 | 8,2 | 9,1 |
|  | Keep my eating habits | 6,5 | 7,6 | 6,4 | 6,8 | 5,0 | 7,4 | 4,6 | 5,4 |
|  | Natural fibre | 9,6 | 10,3 | 9,2 | 11,7 | 8,3 | 10,3 | 9,6 | 7,6 |
|  | Nothing else | 17,3 | 15,8 | 23,8 | 11,0 | 26,3 | 9,9 | 19,9 | 19,0 |
|  | Present in varied products | 12,8 | 11,2 | 12,9 | 13,4 | 12,1 | 12,8 | 11,4 | 14,2 |
|  | Same price | 9,5 | 9,6 | 6,9 | 10,4 | 7,6 | 11,9 | 9,7 | 10,6 |
|  | Same taste, texture, colour | 8,1 | 7,0 | 7,3 | 8,8 | 7,8 | 9,7 | 7,0 | 8,6 |

**Table S7**: Socio-demographic characteristics of the whole sample and for each group of respondents, identified through hierarchical clustering. The five last columns provide the difference between each cluster and the mean surveyed population; when this difference exceeds 20%, cells are highlighted in green (the cluster gives this answer more often than the mean population) or red (the cluster gives this answer less often than the mean population).
